# Hypocomplementemic Urticarial Vasculitis: A Case Report

**DOI:** 10.7759/cureus.100143

**Published:** 2025-12-26

**Authors:** Elisa E Aparicio, Diana V Guerrero, Valerie D Alcántara, Salvador A Gutiérrez, Gabriela R Arce

**Affiliations:** 1 Dermatology, ISSSTE General Hospital Tacuba, Mexico City, MEX

**Keywords:** hypocomplementemic urticarial vasculitis, hypocomplementemic urticarial vasculitis syndrome, urticaria, urticarial vasculitis, vasculitis

## Abstract

Urticarial vasculitis (UV) is an inflammatory condition that affects small vessels, generating urticarial lesions with wheals lasting >24 hours. It can be divided into two main groups: normocomplementemic (NUV) and hypocomplementemic urticarial vasculitis (HUV). The latter is a rare condition, whose association with autoimmune diseases, primarily systemic lupus erythematosus (SLE), makes its diagnosis difficult. We present a 67-year-old female patient with a family history of SLE. She presented with disseminated dermatosis of eight months' duration with wheals lasting >24 hours. Due to a suspected diagnosis of UV, laboratory studies and a skin biopsy were performed, revealing a predominantly neutrophilic perivascular infiltrate, nuclear dust, and mild fibrinoid necrosis, as well as low serum complement levels. Meanwhile, anti-Smith, anti-double-stranded DNA, anti-Ro/SSA, and anti-La/SSB antibodies were all negative. A low-titer ANA (1:100) was detected, although this nonspecific finding is common in healthy individuals and lacks diagnostic significance. Anti-C1q antibodies could not be assessed due to unavailability within the institution. Despite this limitation, the constellation of clinical, laboratory, and histopathological findings supported the diagnosis of HUV, and a favorable therapeutic response was achieved with prednisone at a dose of 0.5 mg/kg per day with a weekly taper of 5 mg until discontinuation, accompanied by close clinical monitoring, including quarterly dermatologic evaluations and semiannual rheumatologic assessments supported by laboratory testing. This case highlights the importance of early identification, the need for a multidisciplinary approach, as well as the exclusion of systemic involvement while ensuring close follow-up, as a high percentage of patients may develop SLE.

## Introduction

Urticaria is a common condition observed in the global population. Urticarial vasculitis (UV) accounts for approximately 10% of all patients with urticaria; clinically, it presents with wheals lasting more than 24 hours, which may leave purpuric or post-inflammatory residual macules upon resolution and are often accompanied by pain or pruritus. In some cases, lesions may persist for several days, and angioedema may also occur [1,2]. According to the revised vasculitis nomenclature and the dermatologic appendix of the 2012 Chapel Hill Consensus Conference, UV can manifest in two forms: normocomplementemic urticarial vasculitis (NUV) and hypocomplementemic urticarial vasculitis (HUV), with the former representing up to 80% of cases. The remaining corresponds to HUV, a small-vessel vasculitis triggered by the formation of immune complexes generated when anti-C1q antibodies bind to the collagen-like region of C1q. This interaction results in endothelial deposition of autoimmune complexes, activation of the classical complement pathway, release of anaphylatoxins, mast-cell degranulation, and subsequent recruitment and activation of neutrophils and eosinophils. Together, these events lead to vascular injury and may produce organ involvement, most commonly affecting the skin, eyes, lungs, kidneys, gastrointestinal tract, and musculoskeletal system. This condition is associated in up to half of cases with underlying autoimmune diseases, most commonly systemic lupus erythematosus (SLE). This association creates diagnostic challenges due to the similarity between the two conditions and, therefore, highlights the need for a multidisciplinary team for proper evaluation, treatment, and follow-up [1,3].

## Case presentation

**Figure 1:**
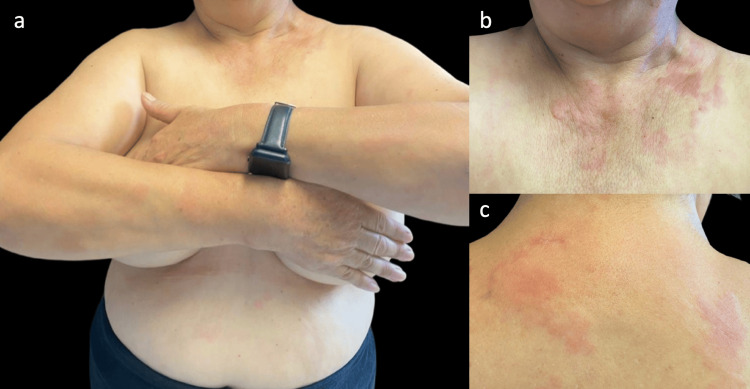
Cutaneous manifestations of hypocomplementemic urticarial vasculitis Patient with an eight-month evolution disseminated dermatosis characterized by multiple wheals involving the décolleté region, upper extremities, epigastrium (a)(b), paravertebral area, and supra-scapular region (c).

The patient is a 67-year-old woman with a family history of SLE and a personal history of osteoporosis treated with calcium and vitamin D. The patient initially presented to the rheumatology department with a history of transient, migratory arthralgias affecting the wrists, elbows, and knees. On physical examination, the rheumatologist also identified a “generalized dermatosis," prompting referral to our dermatology department. At the time of evaluation, both the cutaneous manifestations and arthralgias had been present for eight months. During the physical examination, we observed a disseminated dermatosis on the upper extremities involving the lateral aspects of both arms, the décolleté area, the upper third of the back, and the epigastrium. The dermatosis was characterized by multiple wheals of variable sizes (the largest measuring 6 cm), pale pink, with a smooth surface, well-defined borders, and soft consistency. These lesions caused intense pruritus and typically lasted more than 24 hours (Figure 1).

**Figure 2:**
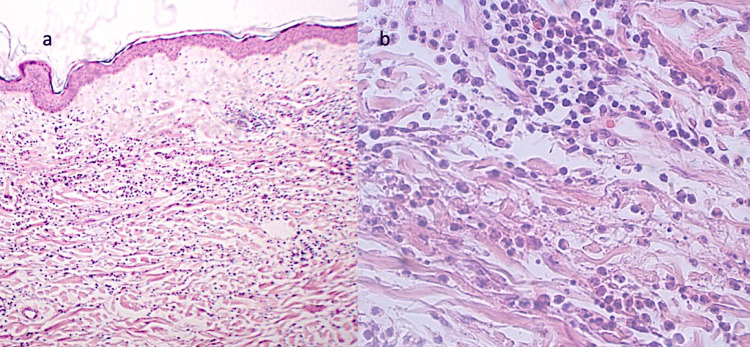
Histological examination of the affected skin Skin biopsy (H&E stain) demonstrates edema in the superficial dermis with a mixed perivascular infiltrate extending into the reticular dermis (panel a, 10×), with a predominance of neutrophils, eosinophils, and marked nuclear dust (panel b, 200×).

The biopsy revealed a perivascular mixed infiltrate with a predominance of neutrophils and eosinophils in the superficial and reticular dermis, along with abundant nuclear dust and mild fibrinoid necrosis (Figure 2). Laboratory studies, performed as part of the diagnostic workup, demonstrated reduced C3 and C4 levels, as well as elevated C-reactive protein (CRP) and thyroid-stimulating hormone (TSH) (Table 1).

**Table 1:** Laboratory findings RBC: red blood cells; ESR: erythrocyte sedimentation rate; CRP: C-reactive protein; RF: rheumatoid factor; Anti-CCP: anti-cyclic citrullinated peptide; anti-Sm: anti-smith antibody; Anti-dsDNA: anti-double stranded DNA antibodies; ANA: antinuclear antibody; anti-Ro/SSA: anti-Sjögren's syndrome-related antigen A; anti-La/SSB: anti-Sjögren's syndrome-related antigen B; TSH: thyroid stimulating hormone;*: abnormal values

Laboratory test	Patient's value	Reference range
Hemoglobin (g/dL)	13	12-15.5
Hematocrit (%)	44	36-47
Platelets (/µL)	298,000	150,000-450,000
Leukocytes (×10³/µL)	5.52	4-11
Glucose (mg/dL)	101*	70-100
Creatinine (mg/dL)	0.9	0.6-1.3
Urinalysis	RBC: 2/HPF	RBC: 0-3/HPF
ESR (mm/hr)	40*	<20
CRP (mg/L)	12.7*	<5
RF (IU/mL)	7	<14
Anti-CCP (U/mL)	0.6	<20
Anti-Sm	Negative	Negative
Anti-dsDNA (IU/mL)	0.63	Negative: <1
ANA	1:100 (weak positive)	Negative: <1:80
Anti-Ro/SSA	Negative	Negative
Anti-La/SSB	Negative	Negative
C3 (mg/dL)	53	90-180
C4 (mg/dL)	3	10-40
T4 (µg/dL)	6.88	5-12
TSH (µIU/mL)	10.30*	0.4-4.5

**Figure 3:**
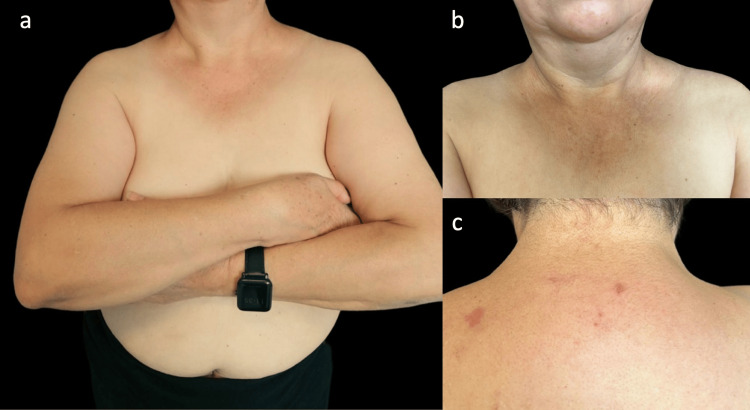
Clinical outcome after corticosteroid administration Panels (a), (b), and (c) show the patient two months after systemic corticosteroid therapy, with near-complete remission of the lesions.

The presence of chronic urticaria persisting for more than six months, alongside hypocomplementemia and histologic findings of leukocytoclastic vasculitis, led to the diagnosis of HUV. Systemic corticosteroid therapy was initiated with prednisone at a dose of 0.5 mg/kg per day with a weekly taper of 5 mg until discontinuation, resulting in near-complete remission of the lesions within two months (Figure 3). Given the patient’s family history, laboratory findings, and the strong association of HUV with autoimmune disorders, a repeat evaluation by the rheumatology department was requested. Systemic involvement was ruled out, and the previously established diagnosis was confirmed. The patient was also referred to the endocrinology service following the identification of subclinical hypothyroidism.

## Discussion

UV is a rare small-vessel vasculitis, reported in approximately 2-27% of patients being followed for urticaria. The condition predominantly affects females, aged 35-51 years [4]. UV may be idiopathic or associated with multiple conditions, including infections (hepatitis B and C viruses, HIV, Epstein-Barr virus, Borrelia burgdorferi, etc.), serum sickness, neoplasms, drugs (cimetidine, fluoxetine, procainamide, atenolol, sulfamethoxazole, paroxetine, ciprofloxacin), and UV exposure, among others. UV can be further classified into NUV and HUV, with the former being more common. The HUV carries a higher risk of association with autoimmune diseases, most frequently SLE, mixed connective tissue disease, rheumatoid arthritis, and juvenile idiopathic arthritis [2,5].

The definitive diagnosis of HUV is based on clinical manifestations, histological findings, and the presence of low complement levels (C3, C4, and anti-C1q antibodies). Histologically, leukocytoclastic vasculitis is characterized by a mixed perivascular inflammatory infiltrate with neutrophilic predominance affecting the walls of capillaries and postcapillary venules, accompanied by nuclear dust (leukocytoclasia) and, in some cases, fibrinoid necrosis. Although anti-C1q antibodies play a central role in the pathophysiology of HUV and are highlighted in the Chapel Hill nomenclature, their measurement was not feasible due to institutional limitations. Nevertheless, the diagnosis of HUV can still be supported when other key clinical, laboratory (hypocomplementemia), and histopathologic features are present. Taken together, these findings provide sufficient grounds to establish HUV despite the unavailability of anti-C1q testing [3,6,7].

HUV may present as an isolated condition, fulfilling the diagnostic criteria described above, or as hypocomplementemic urticarial vasculitis syndrome (HUVS). HUVS results from a type III hypersensitivity reaction, leading to the formation of immune complexes that mediate multiorgan involvement. Clinical manifestations primarily affect the musculoskeletal, pulmonary, renal, ocular, and gastrointestinal systems [8]. The most frequent manifestation is arthralgia or arthritis, predominantly involving the hands, elbows, knees, and ankles, observed in 50-92% of cases [7,9].

The diagnosis of HUVS is supported by the Schwarz Criteria, which consider the presence of two major criteria (chronic urticaria lasting more than six months and hypocomplementemia) and two minor criteria (leukocytoclastic vasculitis on skin biopsy, arthralgia or arthritis, glomerulonephritis, uveitis or episcleritis, recurrent abdominal pain, low C1q with positive anti-C1q antibodies). In the case of the patient presented, both major criteria were met, along with the presence of arthralgias and leukocytoclastic vasculitis on skin biopsy, fulfilling the minor criteria [1,10].

Patients with HUV should undergo complementary testing aimed at identifying potential underlying causes and associated autoimmune diseases. Recommended studies include: complete blood count, serum chemistry panel, urinalysis, renal function tests, C-reactive protein, erythrocyte sedimentation rate, rheumatoid factor, antinuclear antibodies, anti-dsDNA, anti-Ro/SSA, anti-La/SSB, antiphospholipid antibodies, antineutrophil cytoplasmic antibodies (ANCA), antithyroid antibodies, cryoglobulins/cryoﬁbrinogen, serologies for hepatitis A, B, and C viruses, *Borrelia* species, *Mycoplasma pneumoniae*, Epstein-Barr virus (including viral load); anti-DNase B; as well as chest radiography, pulmonary function tests, echocardiography, imaging to assess joint involvement, and ophthalmologic evaluation (particularly when systemic manifestations are present) [11].

When malignancy is suspected, screening should be performed according to age, sex, and guided by clinical findings and/or abnormalities identified on prior studies. Relevant diagnostic tests may include: serum protein electrophoresis, lactate dehydrogenase, fecal occult blood testing, tumor markers, chest radiography, mammography, abdominal ultrasound, computed tomography, and PET-CT [11,12]. Some ancillary investigations were not obtained due to institutional constraints, as well as the absence of specific clinical indications that would have increased their diagnostic utility. In this patient, the presentation was dominated by cutaneous manifestations; there were no clinical signs suggestive of active viral infection (e.g., jaundice, constitutional symptoms, or relevant hepatic abnormalities), nor features typically associated with cryoglobulinemia, such as severe Raynaud phenomenon, peripheral neuropathy, or distal purpuric ulcerations. Although the lack of certain complementary tests represents a limitation, these omissions reflect real-world practice in resource-restricted settings and were aligned with the clinical scenario at the time of assessment.

SLE is the autoimmune disease most commonly associated with HUV, as both conditions involve immune complex formation and can present with overlapping clinical and laboratory features. Some authors even consider HUV as a variant of SLE. However, there are distinguishing features that may help differentiate between the two entities, including the presence of malar rash, the type of pulmonary involvement (obstructive in HUV versus restrictive in SLE), and laboratory markers such as anti-dsDNA, anti‑Sm, anti‑Ro/SSA, and anti‑La/SSB antibodies, which are more indicative of SLE. A low-titer ANA (1:100) was also detected; however, this finding is nonspecific and may occur in healthy individuals, thus lacking diagnostic significance in this context [1,13].

Despite the patient’s family history and the strong association with SLE, she did not meet the European League Against Rheumatism (EULAR)/American College of Rheumatology (ACR) classification criteria for SLE. Laboratory results, including anti‑Smith, anti‑dsDNA, anti‑Ro (SSA), and anti‑La (SSB) antibodies, were negative, making this diagnosis unlikely. While a definitive relationship with SLE was not established, our patient exhibited subclinical hypothyroidism. Recent studies have reported an association between UV and autoimmune hypothyroidism, suggesting an underlying autoimmune predisposition [14]. Given the patient’s age and elevated TSH, she was referred to the endocrinologist for initiation of thyroid hormone replacement and close monitoring.

Finally, due to the rarity of the disease, no standardized treatment guidelines are available. The most commonly used therapies, and those supported by the strongest evidence, include systemic corticosteroids, dapsone, H1 antihistamines, hydroxychloroquine, nonsteroidal anti-inflammatory drugs (NSAIDs), colchicine, cyclophosphamide, azathioprine, omalizumab, IL-1β/IL-1R inhibitors, methotrexate, and rituximab. Among these, systemic corticosteroids have shown the highest rates of complete and partial remission, followed by dapsone, hydroxychloroquine, and NSAIDs. In the patient presented, systemic corticosteroids were initially administered, resulting in significant clinical improvement. This approach was chosen based on the strong evidence supporting corticosteroids for rapid disease control, while persistent or refractory manifestations may later warrant immunosuppressive or targeted therapies [6,15].

## Conclusions

UV is a rare disease, highlighting the importance of its recognition to improve diagnostic efficiency in clinical practice. This condition can present systemically, affecting the function of multiple organs. The hypocomplementemic form of UV, in particular, is frequently associated with autoimmune diseases, such as SLE. This association can complicate the diagnostic process, as both conditions share similar clinical features.

Understanding the differences in clinical manifestations and laboratory findings between UV and SLE is essential for guiding an accurate diagnosis. Recognizing the association with autoimmune diseases enables a targeted evaluation for these conditions. Timely diagnosis and intervention are critical; prompt identification of HUV is crucial to prevent severe complications, predominantly arthritis resulting in joint damage; membranoproliferative or mesangioproliferative glomerulonephritis, which can progress to renal failure; difficult-to-treat chronic obstructive pulmonary disease; laryngeal edema; episcleritis; uveitis; and intestinal ischemia. For these reasons, close follow-up is essential. Therefore, dermatologic evaluations will be conducted quarterly, while rheumatology and endocrinology visits will be conducted semiannually, supported by routine laboratory testing, including blood chemistry, complete blood count, periodic complement levels, urinalysis, renal function tests, and thyroid function tests, to enable early detection of organ involvement and monitoring of previously identified comorbidities. If clinical findings suggest specific organ involvement, additional studies would be obtained, and referral to the corresponding specialty would be requested (e.g., ophthalmology, nephrology, gastroenterology, and pulmonology).

In summary, sustained clinical vigilance and coordinated care are key elements in managing HUV effectively and reducing the risk of long-term organ damage.
